# Computer acquisition and analysis of data in enzymatic-fluorimetric-continuous-flow methods for the measurement of glucose, lactate, pyruvate, alanine, glycerol and 3-hydroxybutyrate in human blood

**DOI:** 10.1155/S1463924686000123

**Published:** 1986

**Authors:** Clive S. Hetherington, Michael Keir, Raymond Stappenbeck, Alistair Simm, Lindsay L. Brigham, Andrew W. Skillen, Alan W. Hodson

**Affiliations:** 1 University Department of Clinical Biochemistry and Metabolic Medicine Royal Victoria Infirmary Newcastle-upon- Tyne UK; 2 Department of Medical Physics Royal Victoria Infirmary Newcastle-upon- Tyne UK


Journal of Automatic Chemistry/*Journal of Clinical Laboratory Automation*, Vol. 8, No. 2 (April–June 1986), pp. 49–55

### Computer acquisition and analysis of data in enzymatic-fluorimetric-continuous-flow methods for the measurement of glucose, lactate, pyruvate, alanine, glycerol and 3-hydroxybutyrate in human blood

**Clive S. Hetherington, Michael Keir*, Raymond Stappenbeck, Alistair Simm, Lindsay L. Brigham, Andrew W. Skillen and Alan W. Hodson†**

*University Department of Clinical Biochemistry and Metabolic Medicine, Royal Victoria Infirmary, Newcastle-upon-Tyne, UK*

### Introduction

Enzymatic-continuous-flow methods, essentially similar to those previously described [1], are used by the authors to measure metabolites of carbohydrate metabolism in blood after protein precipitation and stabilization with perchloric acid. These techniques, combined with fluorimetry, allow up to 140 samples, for three metabolites, to be analysed each day. The precision obtained and the cost per sample compare very favourably with the tedious manual spectrophotometric methods.

Drawing calibration graphs with a series of standards and comparing large numbers of peaks is necessarily time-consuming and a notorious source of errors in continuous-flow-analysis. With the authors' methods, moreover, the problems are virtually doubled because two autoanalyser 'runs' are made for each metabolite, one of which is needed to determine the intrinsic or 'blank' fluorescence present in the perchloric acid extracts of blood and the other for the 'test' run. These extracts are prepared by adding an approximate volume of blood to perchloric acid and although this allows some flexibility at the blood collection stage, a different factor has to be applied to each sample to correct for the dilution obtained and is, therefore, additionally time-consuming. For these reasons a means of acquiring the analytical data from the fluorimeters using a microcomputer has been devised, thus lessening very much the tedium of the analyst, as well as possibly eliminating many of the calculation errors.

### Methods and materials

The instrumental system consists of a Chemlab sampler type CS40-80 (Chemical Instruments Ltd, Hornminster House, 129 Upminster Road, Hornchurch, Essex RM11 3XJ, UK) and a Technicon AA2 proportioning pump (Technicon Instruments Ltd, Hamilton Close, Houndsmill, Basingstoke, UK) which divide the samples into three streams. Each of these is mixed first with the reaction buffer and then with the appropriate enzyme and coenzyme solution. Afterwards the reaction is allowed to proceed for a requisite time in mixing coils and the resultant change in NADH concentration is measured in three Perkin-Elmer fluorimeters, types 1000 or 1000M (Perkin-Elmer Ltd, Post Office Lane, Beaconsfield, Buckinghamshire HP9 1QA, UK). A permanent record of the voltage changes on the LED displays of the fluorimeters is obtained on a Kipp & Zonen four-channel potentiometric recorder, type BD101 (Kipp & Zonen, Delft, Holland, PO Box 507, 2600 AM Delft, The Netherlands). The digital signals from the fluorimeter LED displays are also inputed by a parallel line interface to an Apple II 48K computer (Apple Computers Inc., 10260 Bandley Drive, Cupentino, California 95014, USA). The other components of the data processing equipment are two Apple floppy disk drives, a compatible VDU and an Epson MX-82 dot matrix printer (Epson UK Ltd, Dorland House, 388 High Road, Wembley, Middlesex HA9 6UH, UK). As the computer memory is inadequate to acquire data from the fluorimeters and simultaneously compute the concentrations of the metabolites in the blood samples the latter can only be done after the continuous-flow analytical process has ended or by transferring the data disk to a duplicate microcomputer system.

Some modifications were made in the original continuous-flow techniques [1] mainly concerning the buffers, the concentrations of various enzymes, coenzymes and the concentration range of the working standards used. These are as follows:

*Glycerol:* Buffer: 0·2 moles/l glycine, 1 mole/l hydrazine hydrate, 0·01 mole/l $MgCl_2$, brought to pH 9·6 with 1·0 mole/l NaOH. Enzyme–coenzyme reagent: 2 mg NAD, 2 mg ATP, 17 U glycerol-3-phosphate dehydrogenase, 2·1 U glycerokinase per ml 0·4 moles/l triethanolamine buffer pH 7·4. Working standards: 20–120 μmoles/l.

*3-Hydroxybutyrate:* Buffer: 0·1 mole/l TRIS, 1·3 mmoles/l EDTA brought to pH 8·5 with 1·0 mole/l HCl. Enzyme-coenzyme reagent: 2 mg NAD, 1 U 3-hydroxybutyrate dehydrogenase per ml 0·1 mole/l phosphate buffer pH, 7·4. Working standards: 20–120 μmoles/l.

* Department of Medical Physics, Royal Victoria Infirmary.
† Correspondence should be addressed to Dr Hodson.

*Alanine:* Buffer: 0·04 mole/l TRIS, 1 mole/l hydrazine, 1·3 mmoles/l EDTA brought to pH 10·0 with 1·0 mole/l HCl. Enzyme–coenzyme reagent: 2 mg NAD, 1·5 U alanine dehydrogenase per ml 0·1 mole/l phosphate buffer, pH 7·4. Working standards: 50–300 μmoles/l.

*Lactate:* Buffer: 0·5 Mole/l glycine, 0·2 mole/l hydrazine hdyrate, 5·2 mmoles/l EDTA brought to pH 9·6 with 1·0 mole/l NaOH. Enzyme–coenzyme reagent: 2 mg NAD, 30 U lactate dehydrogenase per ml 0·1 mole/l phosphate buffer pH 7·4. Working standards: 200–120 μmoles/l.

*Glucose:* Buffer: 0·1 mole/l triethanolamine 2 mmoles/l $MgCl_2$, brought to pH 8·0 with 1·0 mole/l HCl. Enzyme–coenzyme reagent: 3·2 mg NADP, 3·7 mg ATP, 7 U glucose-6-phosphate dehydrogenase, 14 U hexokinase per ml 0·4 mole/l triethanolamine-HCl pH 7·4. Working standards: 500–3000 μmoles/l.

*Pyruvate:* Buffer: 0·4 mole/l triethanolamine, 10 mmoles/l EDTA, brought to pH 7·4 with 1·0 mole/l HCl. Enzyme–coenzyme reagent: 0·055 mg NADH, 2 U lactate dehydrogenase per ml 0·1 mole/l phosphate buffer, pH 7·4. Working standards: 10–60 μmoles/l.

Other modifications are that the blood samples are collected into 5% w/w perchloric acid (approximately 0·77 mole/l). The standards are prepared in 3% w/w perchloric acid (approximately 0·46 mole/l), and this solution is used as a wash fluid between samples in the flow system and for further dilutions of the blood extracts when necessary. These solutions were more convenient to prepare from the stock 60% w/w solution supplied by the manufacturer (BDH) than the 0·8 mole/l and 0·5 mole/l solutions used in the original method.

### Computer program

The software package put together to process the fluorimeter and other data is made up of four subprograms written in Basic for the Apple II microcomputer. A flow chart of the program is shown in figure 1 and a copy of the whole listing can be obtained from the authors.

### *Acquisition*

This program allows the output from up to three different flow channels to be taken simultaneously, each of which must be identified separately by the analyst who is also responsible for starting and stopping the acquisition of data from each channel at the correct time.

The start of data acquisition is signalled using the fluorescence peak of a high standard just as this begins to register on the fluorimeter LED display. At the sampling rate used about 20 data points per peak are obtained, and the system can cope with up to 140 blood extracts, their associated controls, washes and standards. Correct functioning requires a minimum number of 60 samples.

### *Amend*

With the amend subprogram the analyst can display on the VDU the fluorimeter data in groups of 25 peaks very similar to that traced by the potentiometer recorder. This is one of the variety of options available whereby the data can be examined and edited as necessary, for example, to alter the base-line, to remove or insert peaks and to redraw peaks which have been deformed. In general, it is possible to change anything in the data or correct mistakes in the sequence of samples, standards etc., which might interfere with the analysis subprogram.

### *Analysis*

This subprogram converts the data from the fluorimeters into concentrations. A mandatory requirement is that the sampler turntable is loaded with a set squence of samples and standards (Stds). The following sequence is used: Std 6—Wash—Wash—Std 6—[these Stds are the 'starts'; the first is used to set the fluorimeter and recorder, the second is used as a check and to start the computer data acquisition]— Wash—Wash—*Std 1—Std 2—Std 3—Std 4—Std 5—Std 6—Wash—Low Control—Wash—High Control—Wash—10 samples—Wash—10 samples—Wash*. The sequence between the asterisks is repeated continuously until all the sample cups in the batch have been sampled. For the 'blank' run the following sequence must be used: Reference—Wash—Wash—Reference—Wash—Wash—Low Control—Wash—High Control—Wash—Reference—Wash—*10 samples—Wash—Reference—Wash*. Again the sequence between the asterisks is repeated until the whole batch has been sampled. A 'Reference' is a sample of urine diluted approximately 1 in 100 with 3% w/w perchloric acid, and is used to indicate the beginning of a run of 'blanks' and to separate groups of them to aid the computer program to orientate precisely at least at every tenth 'blank'. These references are stored at −20 °C in 10 ml lots.

The analysis program uses peak height to determine the relationship between standards and blood extract samples. The sequence for determining the concentration in the blood extract samples is as follows: (1) fluorimeter data loaded, (2) peaks located and their height determined, (3) standard data printed with precision information, (4) standards appraised for use together, or as separate groups, (5) concentrations in samples calculated, (6) fluorimeter data for 'blank' run loaded, (7) 'blank' peaks located and heights determined, (8) apparent concentration of 'blanks' determined, (9) print-out, and (10) the actual concentrations (sample minus the blank) are stored on disk.

Figure 2 shows a typical print-out of data processed by the analysis program. Base-line information indicates the possibility of drift and shows if the data has been analysed in the correct sequence and that spurious peaks have not been included. An examination of the base-line values through the batch indicates whether or not drift has occurred. Standard information shows the values for each set and batch of standards. A set is all the standards at a particular concentration. A batch is a series of the standards used preceding a group of blood extract samples. The mean, mean deviation and standard deviation are given for each set of standards. The mean deviation is a positive value and is the average difference

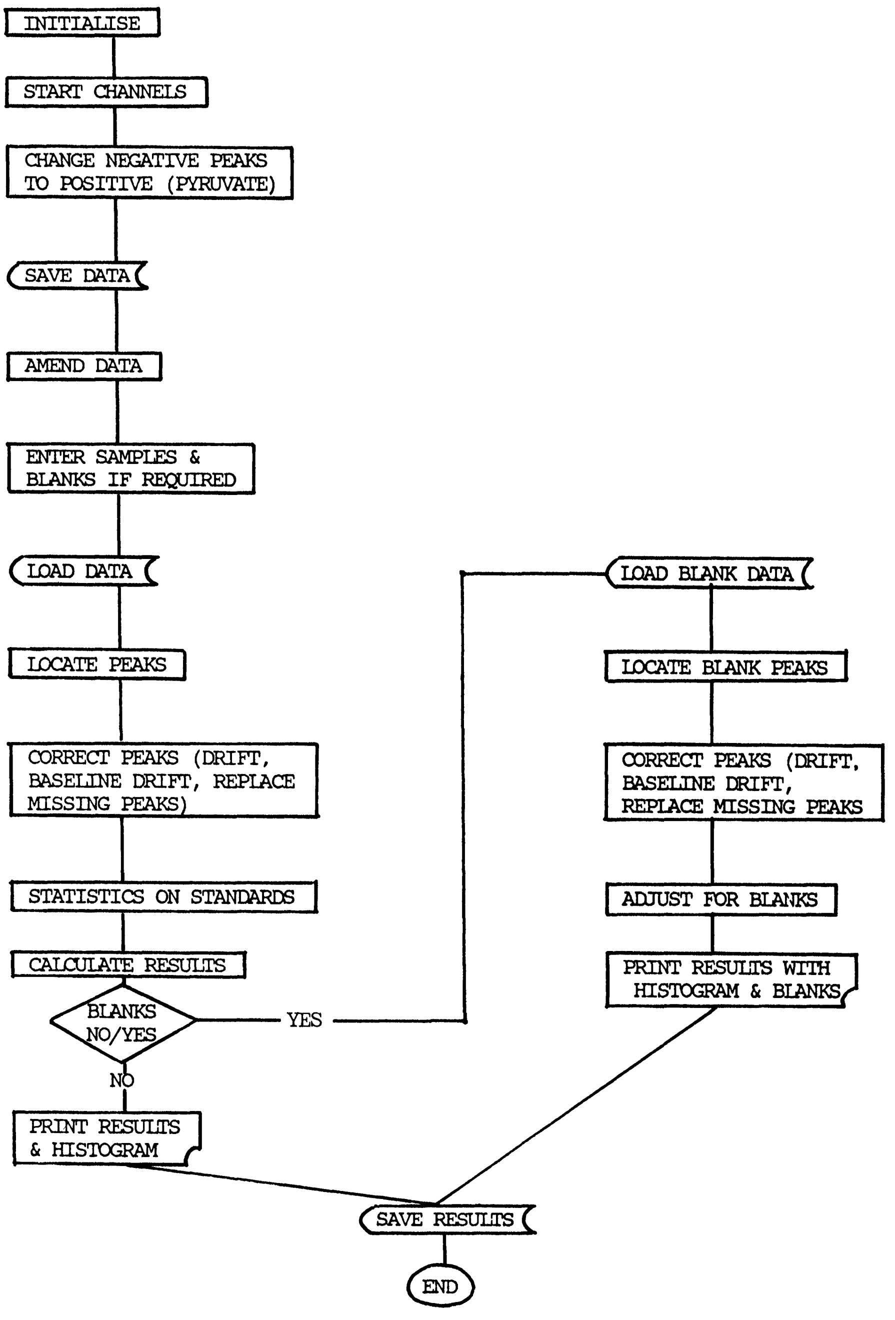

*Figure 1. Flow chart of the program.*

between the mean of the standards and each individual standard. The statistical standard deviations (SD) will not be different from the mean deviation (MD) if there is little difference in the standards. If one standard is very much different from the others in a set the SD is very much greater than the MD. When these differ by more than 25% or the MD is greater than 1·0% of the mean, all the standards cannot be taken together to determine the concentrations of the blood extract samples. The whole group of standards, however, are accepted if the SD for the lowest standard is less than 0·8. Thereafter, the increment must not be more than 0·1 for each step in the concentration of the standards up to an MD of 1·4 for the highest standard. The correlation coefficient, slope and mean printed below each batch of standards also gives useful information. The correlation coefficient indicates the degree of curvature in the relationship of peak height to concentration. Both the correlation coefficient and slope represent lines of 'best fit'. The lower the value of the correlation coefficient, the greater the curvature and a value less than 0·985 is unacceptable resulting in rejection of that batch. The mean and slope values can indicate a blockage in the flow system or a faulty peak. If the differences between the standards within their sets are small, all the standards will be used to determine the concentrations in the blood extract samples. If there is too much difference among the standards, the concentration of the blood extract samples will be calculated from the preceding and succeeding batch of standards, providing there is not too much difference between these two batches of standards. Should there be unacceptable differences, only the preceding batch of standards is used.

```

RESULTS FOR BOH 9/7/85 RESULTS FOR BOH 9/7/85 RESULTS FOR BOH 9/7/85

BASE VALUE INFORMATION.

------------------------

BATCH   BEFORE  AFTER   AFTER   AFTER   BETWEEN AFTER

NUMBER  STDS    STDS    QC-1    QC-2    SAMPLES SAMPLES

  1     81      84      81      83      81      82

  2     82      84      81      82      83      83

  3     83      85      83      84      82      83

  4     83      84      82      85      82      83

  5     83      86      82      84      83      83

  6     83      85      83      84      83      87

  7     87      86      82      83      81      146

STANDARDS INFORMATION.

------------------------

STANDARD                BATCH STANDARD NUMBER                    MEAN    MEAN     STD

 CONC'N   1      2      3      4      5      6      7      8             DEV'N    DEV'N

-------+------+------+------+------+------+------+------+------+-------+--------+-------

   1     53     55     54     55     57     58     55     234    77.6    39.094   63.205

   2     118    109    110    113    114    118    112    308    137.8   42.563   68.87

   3     165    167    166    168    174    177    169    135    165.1   7.563    12.845

   4     218    216    218    223    229    233    224    199    220     7.25     10.282

   5     276    272    275    284    284    296    282    265    279.3   7.25     9.392

   6     324    326    331    335    341    349    344    336    335.8   6.75     8.681

COR COEF.999    1      1      1      1      1      1      .646

SLOPE    2.71   2.71   2.76   2.81   2.84   2.93   2.85   1.72   2.666   .2374    .3907

MEAN     164.9  163.6  164.9  168.3  171.3  175.9  169.4  211

 THERE IS TOO MUCH VARIATION BETWEEN THE SETS OF STANDARDS TO USE THE DATA
 COLLECTIVELY. THE VARIATION BETWEEN STANDARD PAIRS WILL NOW BE CHECKED.

BATCH 1 STANDARD  1

BATCH 2 STANDARDS 2 AND 3 USED

BATCH 3 STANDARD  3

BATCH 4 STANDARD  4

BATCH 5 STANDARD  5

BATCH 6 STANDARD  6

BATCH 7 STANDARD  7

                         BLANK DATA
                         ==========

         BATCH          BASELINE  PEAK VALUE  BASELINE   REF.CONC.

           1               0         322         70        .0896

           2               64        324         70        .0904

           3               65        326         70        .091

           4               65        328         70        .0918

           5               66        329         71        .0918

           6               66        330         70        .0924

           7               67        331         70        .0928

           8               67        333         70        .0934

           9               67        334         71        .0934

           10              66        339         70        .0956

           11              67        335         71        .0938

           12              69        339         71        .0952

           13              68        340         72        .0952

           14              65        343         72        .0962

           15              0         0           0         0

MEAN REFERENCE CONC : .0868

 RESULTS FOR BATCH NUMBER 1

SAMPLE       MILLIMOLAR CONCENTRATIONS

NUMBER     SAMPLE      BLANK       ACTUAL      +    +    +    +    +    +    +    +

 QC-1      0.0200      0.0000      0.0200                                      £££££

 QC-2      0.0982      0.0000      0.0982                  £££££££££££££££££££££££££

                                              +    +    +    +    +    +    +    +

  1        0.0284      0.0160      0.0124                                    £££****

  2        0.1142      0.0146      0.0996              £££££££££££££££££££££££££***

  3        0.0136      0.0066      0.0070                                        ££*

  4        0.0174      0.0080      0.0094                                       ££**

  5        0.0150      0.0058 = ·  0.0092                                        ££*

  6        0.0150      0.0070      0.0080                                        ££*

  7        0.0116      0.0070      0.0046                                         £*

  8        0.0084      0.0072      0.0012                                         £*

  9        0.0072      0.0070      0.0002                                          *

  10       0.0080      0.0072:=>   0.0008                                         £*

                                              +    +    +    +    +    +    +    +

  11       0.0090      0.0072      0.0018                                         £*

  12       0.0110      0.0090      0.0020                                         **

  13       0.0120      0.0106      0.0014                                        £**

  14       0.0106      0.0090    · 0.0016                                         **

  15       0.0540      0.0076 =    0.0464                              ££££££££££££*

  16       0.0216      0.0058      0.0158                                      ££££*

  17       0.0384      0.0080      0.0304                                  £££££££**

  18       0.0276      0.0080      0.0196                                    £££££**

  19       0.0182      0.0070      0.0112                                       £££*

  20       0.0140      0.0090 =    0.0050                                        £**

```

*Figure 2. Print-out of the analysis data.*

For example, the first batch of standards is taken with the second batch and used for the first batch of samples. If the second batch of standards is faulty this is replaced by the first, therefore the first and third batches of standards will be used for the second batch of samples. If the third and fourth batches of standards are acceptable, the mean of these will be used for the third batch of samples. It is possible on rare occasions that the first group of standards could be used with all the samples because all the other batches of standards are unacceptable. For example, when the second batch of standards are faulty they will be replaced by the first batch. Should the third batch of standards also be faulty, they will be replaced by the second batch, which itself has been replaced by the first batch. This sequence of events could be repeated throughout assuming that each batch is faulty, and would happen even if the first batch of standards are faulty. A visual examination of the recorder tracings, however, will show that the results are unacceptable. It is possible that a batch of standards will be used when the peak heights were half those of the preceding batch, providing that the correlation coefficient is acceptable. Again this will be avoided by a visual examination of the recorder tracings and by a check of the standards used for each batch of samples.

The 'blank data' on the print-out gives information concerning the blank washes and 'references' and helps to detect when 'blanks' are registered out of phase, as well as being a test for instability and drift. The base-line values refer to the washes placed before and after the references. The values for the latter are shown under the heading of peak value. The apparent concentrations of the 'blanks' are determined from the final batch of standards as are the reference concentrations (ref. conc.). The 'references' are also the only means of verifying that drift has not occurred without repeating a run with the standards and the appropriate enzymes and coenzymes. Should drift occur the program can apply the appropriate correction to the blank values. Very many of the blood samples contain very little intrinsic fluorescence, therefore a faulty sequence of data for the 'blank' peaks is very likely to occur. The 'reference' peaks are used to overcome this and are easily recognized by their regularity and outstanding height. Between each 'reference' peak there must be 10 'blank' peaks and each of these peaks must be detected within a group of 20 data points. This means that the program can detect the approximate position of each peak and will search for four to five data points on both sides of the anticipated peak to locate it. To facilitate a check of computer results and the potentiometer recorder tracings a histogram is given on the right-hand side of the print-out which gives approximate determined peak heights for both the blanks and tests. The '=' sign in parentheses printed at intervals has no other function than to align the eye along the horizontal lines.

*Collate*

Up to the collate subprogram the data from each of the six metabolite 'runs' is stored separately. In this subprogram there are several options some of which are used to relate the values for each metabolite to its appropriate sample by loading the patient number and relevant sample identification number or order in sequence. Other options are used to include the dilution factor by giving the weights of the collection tubes before and after blood was added. After all the relevant information is collated there are options available to store on disk as a permanent record and print out the original blood values for all the metabolites determined as shown in figure 3.

### Correlation studies

To obtain a correlation [2] between the computer-derived results and those obtained using a chart recorder

METABOLIC ANALYSIS RESULTS 22/5/85

| ASS NO. | LAB NO. | SPL NO. | GLU | 22/5/85 ASSAY CONCENTRATION 22/5/85 LAC | PYR | ALA | GLY | BOH | DILUTION FACTOR | GLU | ADJUSTED CONCENTRATION LAC | PYR | ALA | GLY | BOH |
|---|---|---|---|---|---|---|---|---|---|---|---|---|---|---|---|
| 69 | 4696 | 56 | 1.058 | .128 | .012 | .056 | .009 | .01 | 4.65 | 4.919 | .596 | .057 | .261 | .043 | .046 |
| 70 | ^ | 57 | .775 | .087 | .009 | .041 | .006 | .008 | 6.88 | 5.329 | .599 | .063 | .28 | .042 | .052 |
| 71 | ^ | 58 | 1.109 | .118 | .012 | .056 | .007 | .01 | 5.07 | 5.62 | .597 | .062 | .284 | .037 | .051 |

*Figure 3. Print-out of the calculated blood values.*

and manual comparison of peaks, a batch of approximately 25 samples from five assays to give a total number of 140 were compared. The correlations for the six metabolites were excellent (figure 4 and table 1).

### Discussion

Combining continuous-flow methods with fluorimetry gives a much increased sensitivity over spectrophotometry for the measurement of blood intermediary metabolites of carbohydrate metabolism with a consequential saving in the quantities, and therefore, cost of the enzymes used. There is, however, the disadvantage that two autoanalyser 'runs' must be made, one to determine the concentration of the substrate present, and the other to determine the intrinsic or 'blank' fluorescence in the blood extracts. The concentration of three metabolites in about 140 blood extracts can be assayed in a single day using the flow methods described. Altogether, such a group of samples with their associated standards, controls and 'blank-run' produces something like 1200 peaks to be measured and compared. By devising software for use with an Apple microcomputer, a very much shorter time taken in the calculation of blood metabolite concentrations has been achieved. Another advantage is that computer processing decreases the overall imprecision. Despite specific evidence, this is nevertheless a reasonable assertion, even if only on the grounds that misreading of peak height from a chart and clerical and calculation errors by the analyst are avoided.

The necessity of determining and correcting for 'blank' fluorescence in the blood extracts presents the major difficulty in writing an adequate program for data processing. This problem arises because of the wide range of the 'blank' fluorescence encountered, the importance of which is inversely proportional to both the sensitivity of the method and the concentration of the metabolite. For example, in the case of glucose the 'blank' is negligible and consequently the blank run is omitted. In assays for other metabolites some samples give a 'blank' peak which is very little higher than the variations in the base-line resulting merely from the sample probe passing through air between sample cup and wash fluid. Yet in the same assay with other samples, the blank peak may be higher than the top standard. There is, moreover, the anomaly of the pyruvate method, where a decrease in fluorescence is measured so that the 'blank' value is added. Occasionally, therefore, with pyruvate assays when high blanks are encountered the peaks are lower than the baseline. The program copes with these various demands and particular attention is paid to blank fluorescence.

As far as very low peaks are concerned, it is very difficult to structure a program which is capable of id[illegible]ying and maintaining their correct sequence. This problem is overcome by the presence of a high standard or 'reference' of constant fluorescence placed at set intervals in the 'blank-run'. These 'references', because of their stability, can also be used as a control of the overall sensitivity of the method, especially in relation to the performance of the fluorimeters.

One of the most notable restrictions of the program is the necessity for a rigid sequence of blood extract samples, washes, standards and controls, although for routine purposes this is not entirely disadvantageous. Even so, the 'amend' subprogram does allow post data acquisition flexibility whereby mistakes in the sequence can be corrected by insertion or deletion of data.

By using all the standards together it is possible to smooth out erratic fluctuations in the assay conditions. Alternatively, by using a group of standards, either immediately preceding or succeeding a group of blood extracts, it is

*Table 1. Correlation data.*

| Metabolite | Correlation coefficient A | Correlation coefficient B | *X* intercept A | *X* intercept B | Slope A | Slope B |
|---|---|---|---|---|---|---|
| 3-Hydroxybutyrate | 0·9992 | 0·9553 | −0·00009 | −0·00039 | 1·0183 | 0·9651 |
| Lactate | 0·9906 | 0·9760 | −0·00622 | −0·00207 | 0·9858 | 1·0189 |
| Glucose | 0·9993 | 0·9638 | 0·02229 | 0·021106 | 1·0288 | 0·9839 |
| Glycerol | 0·9630 | 0·9561 | −0·00012 | 0·000011 | 1·0493 | 1·0704 |
| Alanine | 0·9912 | 0·9854 | −0·003501 | −0·00332 | 0·9175 | 0·9494 |
| Pyruvate | 0·9602 | 0·8834 | 0·000090 | 0·00068 | 0·9496 | 1·0802 |

A columns, full standard range.
B columns, zero to lowest standard.

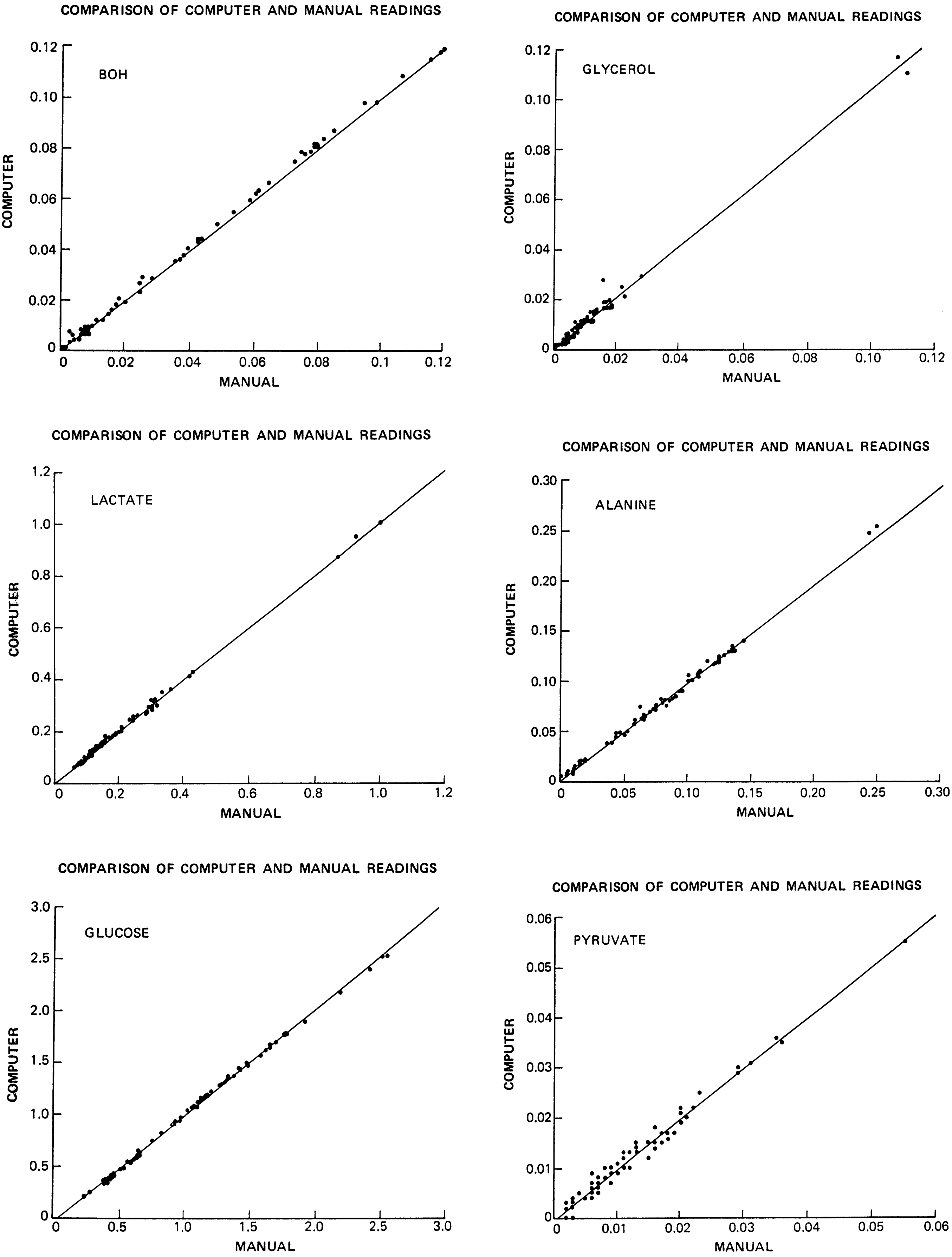

*Figure 4. Comparison between the metabolite concentrations in perchloric acid as determined by manual and computer measurement of peak height.*

possible to allow for base-line drift caused by minor partial blockages in the flow system or changes in temperature that might occur during an assay, which can continue for over 3 h.

Care has been taken to write into the program a means to disregard the spurious peaks resulting from bubbles in the flow system or other unavoidable causes. In spite of these precautions there are occasions when these are accepted by the program as an integral part of the peak. For these reasons, we have retained the potentiometer recorder to identify spurious peaks as well as to act as a back-up in the event of component failure or other causes of break-down in the computer system.

Very little modification to the program is required to determine the metabolite values on the basis of peak area, which is generally held to be more accurate than comparison of peak height. Measuring peak height, however, is the only really practical way to compare peaks by manual methods, therefore, in order to obtain a good agreement between the manual and computer methods the program was also based on peak height. Correlation studies showed that very good agreement was achieved. Of particular interest was the correlation of values between zero and the lowest standard compared with the full standard range for each analyte. The correlations were found to be very similar in both cases, which was contrary to the anticipation that a worse correlation with the lower values would be obtained. The lower correlations for glycerol could be explained by the need to increase the signal amplification of the fluorimeter, because of the very low concentrations analysed, with concomitant increase in electrical noise. This means that the recorder peaks are less smoothly drawn than with the other analytes, but can be more easily compensated for in the manual method. There is a relatively high percentage difference of slope for alanine which could possibly be explained by the use of an awkward concentration scale to determine values in the manual method resulting in an unconscious tendency to round off to consistently lower figures. With pyruvate, when very low concentrations coincide with relatively high blanks, the net result for some samples is a small negative value which would be recorded as zero in the manual, but not with the computer method. This, together with a tendency for the operator to round off values inconsistently, could explain the lower correlation found. The computer is not dedicated to the continuous-flow system so it can be used for other purposes, as well as being altogether a relatively inexpensive hardware package. Whilst the software has been developed especially for analyses where 'test' and 'blank-runs' are required, the latter, of course, can be omitted. In general a variety of analyses other than the measurement of the metabolites described could be substituted.

### References

1. Lloyd, B., Burrin, J. M., Smythe, P. and Alberti, K. G. M. M. *Clinical Chemistry*, **24** (1978), p. 1724.
2. Zar, J. H. *Biostatistical Analysis* (2nd edn, Prentice Hall, New Jersey, 1984), pp. 306–327.

**ANALYTICA 86**

*3 to 6 June 1986 at the Munich Trade Fair Centre*

The scientific programme for this international meeting is divided into symposia, posters and the 'Analytica – Forum München' (in this latter sector, exhibiting companies will present papers on developments in industrial research). Topics to be covered include:

Separation methods
 Chromatographic methods, especially TLC/HPTLC, GC, LC/HPLC
 Electrophoretic methods
Combined methods: MS-MS, HPLC-MS, GC-MS, GC-IR, LC-MS, LC-NMR, FT-GC, GC-FTIR
Emission spectroscopy
Bioluminescence, chemiluminescence, fluorimetry
NMR, its application *in vivo*
Radiochemical procedures
Topochemical procedures
Enzymatic analysis
Cell and organ culture based analysis
Dry support reagents including stick tests
Progress in development of reference methods.

*Enquiries about the commercial exhibition and registration to Münchener Messe- und Ausstellungsgesellschaft mbH, Analytica 86, Postfach 12 10 09, D 8000 München 12, FR Germany. Information about scientific contributions from Professor Dr H. Feldman, Inst. für Physiologische Chemie der Universität, Goethestrasse 33, D 8000 München 2, FR Germany.*

